# The role of nutritional state in the relationship between standard metabolic rate and locomotor activity in juvenile white sturgeon (*Acipenser transmontanus*), with implications for anthropogenically altered food webs

**DOI:** 10.1093/conphys/coaf039

**Published:** 2025-06-12

**Authors:** Vanessa K Lo, Matthew J Hansen, Nann A Fangue

**Affiliations:** Department of Wildlife, Fish and Conservation Biology, University of California Davis, 455 Crocker Lane, Davis, CA 95616, USA; Department of Fish Biology, Fisheries and Aquaculture, Leibniz Institute of Freshwater Ecology and Inland Fisheries, Müggelseedamm 310, Berlin, Germany; Department of Wildlife, Fish and Conservation Biology, University of California Davis, 455 Crocker Lane, Davis, CA 95616, USA

**Keywords:** Behaviour, fish, intraspecific variation, ration, temperature

## Abstract

White sturgeon (*Acipenser transmontanus*) are in decline globally, and populations in the Sacramento–San Joaquin River Basin are particularly vulnerable due to habitat impacts, variable recruitment and altered food availability, all of which are exacerbated by climate change. The minimal metabolic expenditure required to maintain homeostasis, termed standard metabolic rate (SMR), is thought to have broad ecological relevance because it correlates with other important measures of metabolic demand and a range of fitness-related behavioural traits. SMR is variable among individuals and this variation may also underlie variation in behaviour. Additionally, SMR has been shown to be phenotypically flexible in the presence of changing food availability. The objective of this study was to assess how nutritional status may affect the relationship between SMR and locomotor activity in juvenile white sturgeon. We reared white sturgeon at 15°C under an optimal feed rate (OFR, 5.3% bodyweight/day) and low feed rate (LFR, 2.6% bodyweight/day) for 6 weeks, measuring SMR and locomotor activity at the 3- and 6-week timepoints. OFR fish were significantly larger than LFR fish at both timepoints, but mass-specific SMR was not significantly different across treatment or time. We found that only fish under the greatest nutritional stress (6 weeks at LFR) showed a significant relationship between SMR and locomotor activity. This is evidence that observable correlations between physiological and behavioural traits may only become apparent under the influence of environmental stressors. As changing climate is projected to impact food web dynamics and food availability, understanding how nutritional state affects physiological and behavioural traits may help to predict how animals respond to future shifts.

## Introduction

White sturgeon (*Acipenser transmontanus*) are anadromous, long-lived and late maturing fish native to the West Coast of North America. They primarily reside in the waterways and estuaries of the Fraser River (British Columbia), Columbia River (Washington and Oregon) and the Sacramento–San Joaquin River Basin (SSJRB) in California. Like most major river systems on the West Coast, the SSJRB suffers from altered flow, temperature and nutrient regimes due to extensive engineering projects, impassable dams and agricultural conversion (Yoshiyama *et al.,* 1998; Brown and Moyle, 2005; Moyle *et al.,* 2013). White sturgeon spawning and larval rearing success in the SSJRB is threatened by this infrastructure as well as climate change because the frequency of high spring and winter outflows and the intensity of drought events are correlated to recruitment levels (Fish, 2010). Additionally, the SSJRB is considered one of the most heavily invaded estuaries in the world, with documented native fish declines and food web consequences that are not fully understood (Cohen and Carlton, 1998; Sommer *et al.,* 2007; Stompe *et al.,* 2020). White sturgeon are currently categorized as a state Species of Special Concern due to habitat impacts and negative predicted population growth rates (Blackburn *et al.,* 2019). A petition for listing as ‘threatened’ under the California Endangered Species Act was passed in June 2024, following consecutive years of harmful algal blooms that led to substantial adult white sturgeon die-off in the San Francisco and San Pablo Bays (California Fish and Game Commission, 2023). As behaviour and physiology are intertwined, a basic understanding of how environmental factors relate to traits in both disciplines is critical for effective white sturgeon management.

Metabolic rate is a commonly measured physiological trait, as it represents the collective biochemical processes by which organisms transform energy to support life functions and is thought to have evolved in close synchrony with traits related to species’ niches and fitness (Brandl *et al.,* 2023). Standard metabolic rate (SMR), the baseline rate of energy usage required to maintain physiological homeostasis, affects fitness-related traits and thus is expected to be under multiple selective pressures (Burton *et al.,* 2011; Killen *et al.,* 2016). SMR is variable within species, yet also repeatable across age and mass in many taxa (Nespolo and Franco, 2007). Within-species variation in SMR was historically considered noise or measurement error but thanks in part to advances in dissolved oxygen measuring technology, is now understood to reflect consistent between-individual differences in physiology (Metcalfe *et al.,* 2016). Additionally, SMR has been shown to exhibit phenotypic flexibility across days to weeks, where an individual fish’s SMR can increase when food availability is increased or decrease when food levels decline (e.g. juvenile brown trout*,*  Auer *et al.,* 2015, 2016a). Phenotypic diversity and flexibility can be indicative of genetic diversity and the ability of a population to cope and adapt to environmental change. Understanding the ecological and behavioural consequences of variability in SMR may help fisheries managers predict future outcomes to climate impacts.

Physiological and behavioural mechanisms are intertwined and collectively affect decision-making in organisms. SMR, e.g. has been correlated with growth (Álvarez and Nicieza, 2005), food availability (Auer *et al.,* 2016b) and behaviours such as aggression and boldness (Metcalfe *et al.,* 1995). However, the link between SMR and behaviour is not clear, and the effect size, directionality and any resulting fitness consequences appear to be context-dependent (Armstrong *et al.,* 2011). One hypothesis explaining some of this variation is that an observable link between SMR and behaviour may depend on the level of stress the animal is experiencing, with the physiological stressor strengthening or revealing a relationship between the two (Killen *et al.,* 2011, 2012, 2013). A common environmental stressor experienced by animals is insufficient nutrition. In European sea bass (*Dicentrarchus labrax*), metabolic rate correlated with risk-taking behaviour, but only in fish that had experienced food deprivation (Killen *et al.,* 2011). Thus, nutritional state may affect the physical state of an organism, leading to altered metabolic budgeting decisions and divergence in behavioural tendencies (Killen *et al.,* 2013).

Changes to water flow, temperature and nutrients in the SSJRB alter food web dynamics, which has led to the decline of some fish populations, termed the pelagic organism decline (POD; Sommer *et al.,* 2007). The impact on white sturgeon is not fully understood, but for sympatric threatened green sturgeon, evidence suggests that river temperature and discharge related to dam releases in the upper Sacramento River reduce the prevalence of preferred zooplankton prey in larval and early juvenile stomach contents (Zarri and Palkovacs, 2019). The relationship between nutritional state and behaviour in juvenile white sturgeon is understudied and may contribute to poor recruitment. Thus, our goal was to determine whether nutritional state causes shifts in SMR or alters the relationship between SMR and locomotor activity. We hypothesized that ration size would affect SMR over time, predicting that the amount of food positively correlates with SMR, as metabolic flexibility may be especially important for somatic growth during the juvenile stage—a key determinant of fitness because of its effects on body size (Sogard, 1997). Additionally, we hypothesized that locomotor activity was linked to individual differences in SMR, predicting that individuals with high SMRs will be more active than those with low SMRs because greater activity is needed to support the added energetic demands of a high SMR (Mueller and Diamond, 2001; Speakman *et al.,* 2004). Lastly, we hypothesized that the relationship between SMR and locomotor activity would be affected by nutritional state, predicting that any correlation between the two would become stronger with greater nutritional stress (Burton *et al.,* 2011; Killen *et al.,* 2011, 2012).

## Materials and Methods

### Experimental animals

White sturgeon embryos were transported from Sterling Caviar Farm (Sacramento, California) to the Center for Aquatic Biology and Aquaculture at UC Davis in June 2018. All fish were from the same cohort and the number of families was unknown. At hatch, 300 larvae were split evenly among six 300-l replicate flow-through well-water tanks and reared at 15°C. Well-water salinity was 0.4 parts per thousand (ppt) and fish were exposed to natural photoperiod conditions for Davis, CA (38.5°N). Since early developmental stages rely on endogenous yolk reserves (Kamler, 2008), fish were not fed until ~14 days post-hatch (dph), although food was provided at ca.12 dph to orient larvae to chemical cues (Van Eenennaam *et al.,* 2012). Once feeding was detected, larvae were fed *ad libitum* with semi-moist commercial Starter Crumble feed (Skretting, USA) and excess uneaten feed and faeces were removed daily. Feed rates were calculated according to optimal feed rate models for white sturgeon (Deng *et al.,* 2003; Lee *et al.,* 2014) using mean wet mass and water temperature and were updated biweekly to account for fish growth and routinely exceeded to ensure *ad libitum* feed availability. All handling, care and experimental procedures used were reviewed and approved by the UC Davis Institutional Animal Care and Use committee (IACUC, protocol #21834).

The experiment was initiated with 50 dph white sturgeon (mean ± SD; 0.74 ± 0.29 g, 5.24 ± 0.67 cm, *n* = 3000) divided evenly among six tanks. Three tanks received an OFR of 5.3% bodyweight/day and three tanks were reduced to an LFR of 2.6% bodyweight/day. Feed rates were updated biweekly to account for fish growth by randomly selecting 15 fish per tank per treatment for length and wet mass measurements. Tank water temperatures were kept at 14.9 ± 0.4°C, monitored using iButton® temperature loggers (model DS1922L, Maxim Integrated Products, Inc., USA) with a sampling rate of 600 s. Respirometry and locomotor activity trials were conducted after 3 and 6 weeks of ration exposure.

### Respirometry

Oxygen consumption—an indirect measure of metabolic rate (*Ṁ*O_2_, mg O_2_ h^−1^)—of individual juvenile white sturgeon was measured using intermittent flow respirometry in an eight-chamber system. Each chamber (146.05 ± 1.25 ml) consisted of a glass jar with a rubber stopper on the end and was sized to limit the movement of fish. The rubber stopper was pierced with two stainless steel tubes fit with high-grade gas-impermeable silicone tubing that provided recirculating and flush water flow via one 8-channel low-flow peristaltic pump (recirculating flow, Model BT100-1 L, Langer Instruments, USA) and one submersible aquarium pump with an eight-channel manifold and check valves to prevent backflow (flush flow). Chambers were submerged in a 78-l water bath with 15°C flow-through well water. Each chamber had an optical oxygen sensor spot (PreSens, Germany) affixed to the inside of the glass chamber wall with silicone glue, which was read through the glass using fibre optic cables and two 4-channel oxygen metres (Witrox 4, Loligo systems, Denmark). The intermittent flow cycle was set such that chambers never fell <80% O_2_ saturation at the end of each measurement. This ensured the fish did not become hypoxic and stressed (Svendsen *et al.,* 2016). Flush and recirculation periods were controlled using Autoresp™ software (Loligo systems, Denmark). For each trial, seven fish were randomly chosen from rearing tanks and placed in isolation for a fasting period of 24 h to ensure digestion and absorption of nutrients from daily feeding would not elevate *Ṁ*O_2_ (Chabot *et al.,* 2016; Lo *et al.,* 2022). Fish were then transferred to respirometers, with one respirometer left empty to measure background bacterial respiration (Svendsen *et al.,* 2016). After a 1-h adjustment period to the respirometer, *Ṁ*O_2_ measurements began and were conducted for the next ~22 h. The respirometry chambers and water bath were cleaned and dried after every trial, and peristaltic pump tubing was bleached, neutralized and rinsed weekly to prevent bacterial build-up on surfaces. Each respirometry chamber’s sensor spot was calibrated individually with oxygen-free distilled water and fully aerated distilled water every 2 weeks.

### Locomotor activity

Locomotor activity was measured in a circular behavioural arena made from flexible 2-mm white PVC sheeting in a 1 × 1.5 m water table, allowing the size of the arena to be scaled to the total length of each fish (8:1, diameter:fish). The entire water table was encircled by curtains to reduce any disturbance to the fish during behavioural trials. Video was recorded using a GoPro situated ~0.5 m above the arena with a linear field of view and captured in 1080p/30 frames s^−1^. Video analyses were conducted using EthoVision® XT 14. Behavioural trials were conducted after the respirometry trial, with fish selected randomly from respirometers. After fish total length was measured, an individual fish was placed in a mesh holding area at the centre of the arena and allowed 5 min to acclimate, which was deemed sufficient via pilot studies and previous white sturgeon behavioural trials (personal communication from Dr Matthew J. Hansen, Leibniz Institute of Freshwater Ecology and Inland Fisheries, Berlin, Germany). The mesh holding area was lifted out of the arena remotely by string to prevent disturbances, and the fish was allowed to explore the arena for 10 min. Locomotor activity was measured as the total distance moved during the 10-min trial divided by the fish’s total length. The water table was drained and cleaned with alcohol after each trial to remove any remaining cues from other fish. After the behavioural trial, sturgeon were euthanized in a lethal solution of tricaine methanesulfonate (0.5 g l^−1^) buffered with sodium bicarbonate (0.42 g l^−1^) and Instant Ocean® sea salt (6.0 g l^−1^), then measured to the nearest 0.01 g and 0.1 cm. In total, *n* = 10 for OFR fish and *n* = 12 for LFR fish at Week 3, and *n* = 13 for OFR fish and *n* = 13 LFR fish at Week 6, for each measurement of SMR, locomotor activity, length and wet mass.

### Data and Statistical Analysis

Metabolic rate was recorded using Autoresp™ software (Loligo Systems, Viborg, Denmark) and data analyses performed using R (version 4.2.1). On average, 50 *Ṁ*O_2_ measurements were collected per fish and values included in analysis were required to have an R^2^ > 0.96, resulting in an average removal of 12.3% of *Ṁ*O_2_ measurements. For each trial, background bacterial respiration was subtracted from individual fish *Ṁ*O_2_ values. *Ṁ*O_2_ was converted to mass-specific values (mg O_2_ g^−1^ h^−1^) and used to calculate SMR following recommendations and R script provided by Chabot *et al.* (2016), which compares eight methods to provide a best estimate of SMR. For our data, SMR estimations were best using mean of the lowest normal distribution (MLND) in 13 fish, and non-parametric quantile regression in 39 fish. The MLND method is an objective statistical approach that represents SMR well when the coefficient of variation (CV_MLND_) is ≤ 5.4, but other methods are recommended if CV_MLND_ > 7. Non-parametric quantile regression allows a percentage of observations set by the user to fall below SMR—in this case, 20%—a cut-off shown to produce SMR estimates not significantly different from values predicted by the MLND method in data sets with a low CV_MLND_ during Chabot *et al.* (2016)’s comparison of methods.

**Figure 1:**
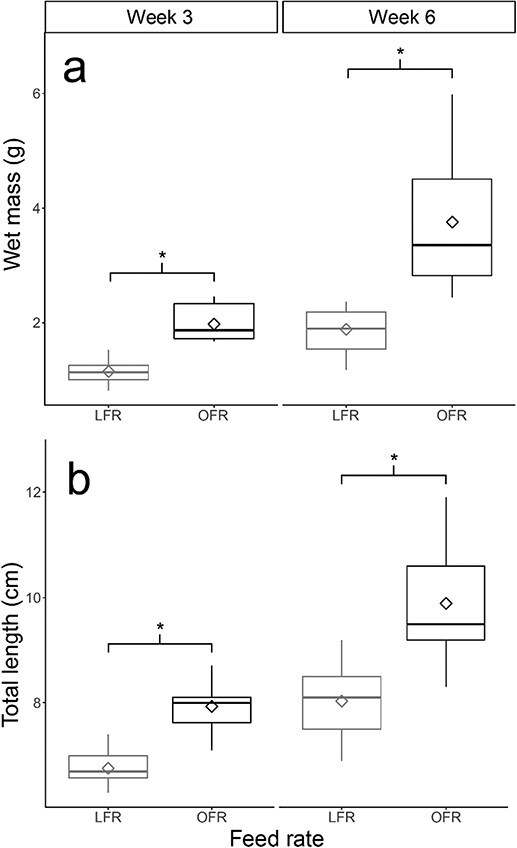
Juvenile white sturgeon (*A. trasmontanus*) growth (**a**: wet mass in grams and **b**: total length in centimetres) at 3 and 6 weeks when reared at 15°C and fed an OFR or an LFR, 50% of OFR). The centreline of the boxplots represents the median, the box represents the interquartile range (IQR), the whiskers extend 1.5 times IQR and diamonds represent the mean. Asterisks indicate significant differences between feed rates at *P <* 0.05.

Tank effects were assessed using a linear mixed-effects model with fish mass as the response variable, week and feed rate as fixed effects and tank as a random effect. However, the model produced a singular variance–covariance matrix for the random effect, likely due to insufficient data per tank. A simplified linear model without tank as a random effect yielded a lower Akaike Information Criterion (AIC), supporting its use. To further evaluate potential tank effects, we analysed tank rearing data where 15 fish were randomly selected and weighed every 2 weeks to track growth under different ration treatments. Wet mass was analysed using a generalized linear mixed model with week, feed rate and their interaction as fixed effects, and tank as a random intercept. A Gamma distribution with a log link function was applied. The estimated variance for the tank intercept (0.00036) was minimal, indicating that tank effects were negligible and unlikely to influence our results. Therefore, most of our subsequent models were found via AIC comparison to have a better fit without the random effect of tank.

Differences in wet mass were modelled using a generalized linear model with week and feed rate as fixed effects with a Gamma distribution and log link function, while total length was modelled using a linear model with week and feed rate as fixed effects. Log-transformed length–weight relationships were modelled with a linear model with week, feed rate and their interaction as fixed effects. SMR and whole-body *Ṁ*O_2_ under each feed rate were compared using Kruskal–Wallis rank sum tests after checking assumptions of normality and heterogeneity of variance. We included the interaction terms of week and feed rate. Pairwise comparisons were conducted using the Wilcoxon rank sum exact test if necessary. Relationships between SMR and locomotor activity were modelled with a linear model with distance travelled, feed rate, their interaction and wet mass as fixed effects. Week 3 trials were analysed separately from Week 6 trials. Results were considered significant at *P* < 0.05.

## Results

**Figure 2:**
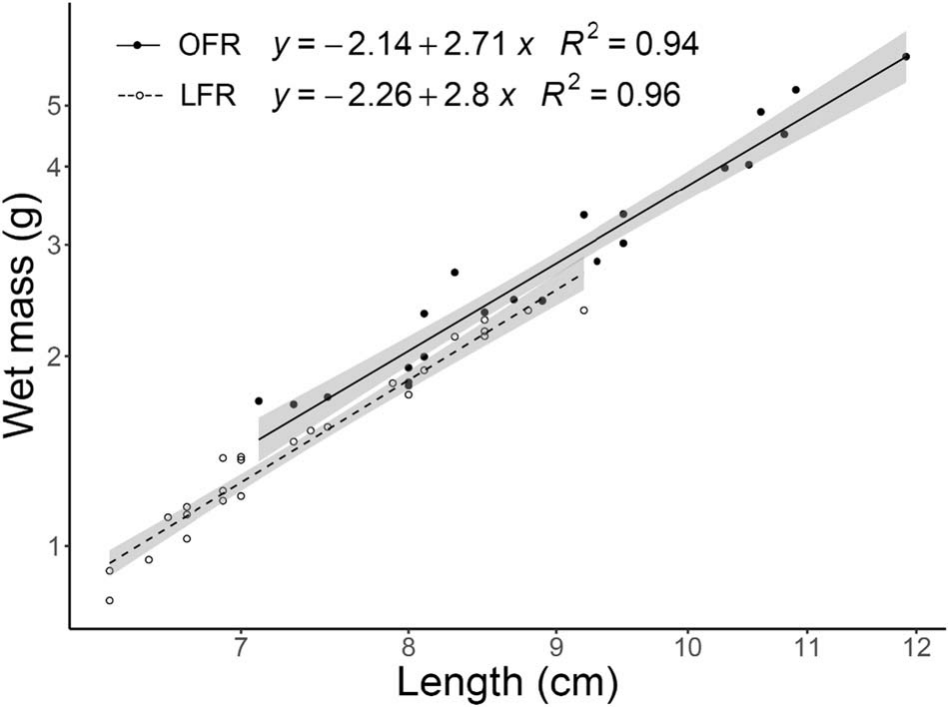
Length–weight relationships of juvenile white sturgeon (*A. transmontanus*) reared at 15°C fed OFR (closed circles and solid line) and LFR (50% of OFR, open circles and dashed line), plotted on log–log transformed axes. Lines represent least squares regressions and grey ribbons represent standard error of the fit.

Mean wet mass (mean ± SD in g; Week 3: OFR = 1.98 ± 0.30, *n* = 10; LFR = 1.15 ± 0.21, *n* = 12; Week 6: OFR = 3.76 ± 1.14, *n* = 13; LFR = 1.89 ± 0.40, *n* = 13) showed a significant positive effect of week (estimate = 0.571, *P* < 0.001) and feed rate (estimate = 0.628, *P* < 0.001) on weight (Fig. 1a). Similarly, mean total length (mean ± SD in cm; Week 3: OFR = 7.9 ± 0.5, LFR = 6.8 ± 0.3; Week 6: OFR = 9.9 ± 1.0, LFR = 8.0 ± 0.7) showed a significant positive effect of week (estimate = 1.639, *P* < 0.001) and feed rate (estimate = 1.554, *P* < 0.001) on total length (Fig. 1b). Mean log-transformed length and wet mass showed a significant positive effect of log-transformed length (estimate = 2.718, *P* < 0.001) and feed rate (estimate = 0.0488, *P* = 0.765) on log-transformed weight. However, the interaction between log-transformed lengths and feed rate was not significant (estimate = −0.0020, *P* = 0.991), indicating that the length–weight relationship did not differ between feed rates (Fig. 2).

**Figure 3:**
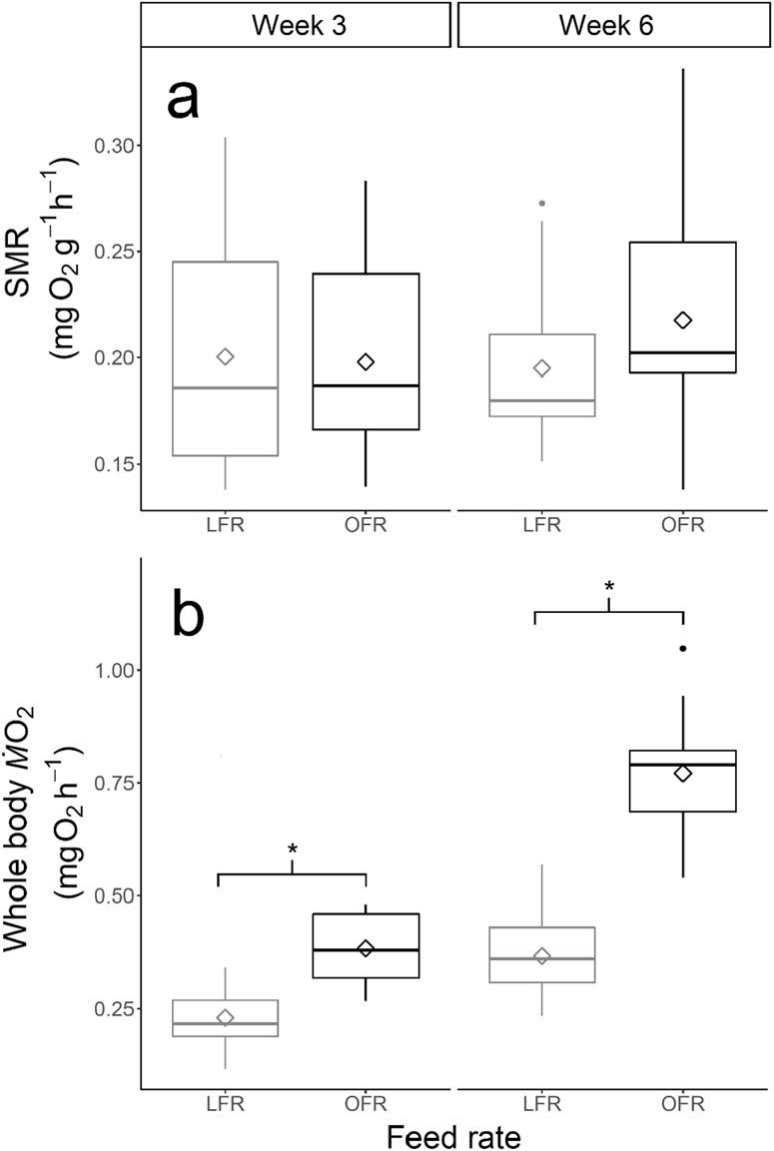
Metabolic rates (**a**: SMR, mg O_2_ g^−1^ h^−1^; **b**: Whole-body metabolic rate, *Ṁ*O_2_, mg O_2_ h^−1^) of juvenile white sturgeon (*A. transmontanus*) reared at 15°C and fed OFR (in black) and LFR (50% of OFR, in grey). The centreline of the boxplots represents the median, the box represents the IQR, the whiskers extend 1.5 times IQR and diamonds represent the mean. Asterisks indicate significant differences between metabolic rates at *P <* 0.05.

SMR, which is mass-specific, showed no significant differences in SMR between feed rates at Week 3 (mean ± SD in mg O_2_ g^−1^ h^−1^; OFR = 0.20 ± 0.05, LFR = 0.20 ± 0.06) or Week 6 (mean ± SD in mg O_2_ g^−1^ h^−1^; OFR = 0.22 ± 0.06, LFR = 0.20 ± 0.04) (chi-squared = 1.8023, *P* = 0.6144) (Fig. 3a). In contrast, the Kruskal–Wallis test revealed a significant difference (chi-squared = 37.154, *df* = 3, *P* < 0.001) in mean whole-body *Ṁ*O_2_ at Week 3 (mean ± SD in mg O_2_ h^−1^; Week 3: OFR = 0.38 ± 0.07, LFR = 0.23 ± 0.07) and Week 6 (mean ± SD in mg O_2_ h^−1^; OFR = 0.77 ± 0.13, LFR = 0.37 ± 0.11). Subsequent pairwise comparisons were all significant except for Week 3 OFR and Week 6 LFR fish (Fig. 3b).

At Week 3, no significant relationship was found between locomotor activity, feed rate or wet mass on SMR (all *P* > 0.05). At Week 6, in contrast, the interaction between locomotor activity and feed rate was significant (estimate = 0.0287, *P* = 0.0007). Additionally, locomotor activity (estimate = −0.0146, *P* = 0.0076), feed rate (estimate = −0.1599, *P* < 0.0001) and wet mass (estimate = −0.0384, *P* = 0.00005) all showed significant negative relationships with SMR.

**Figure 4:**
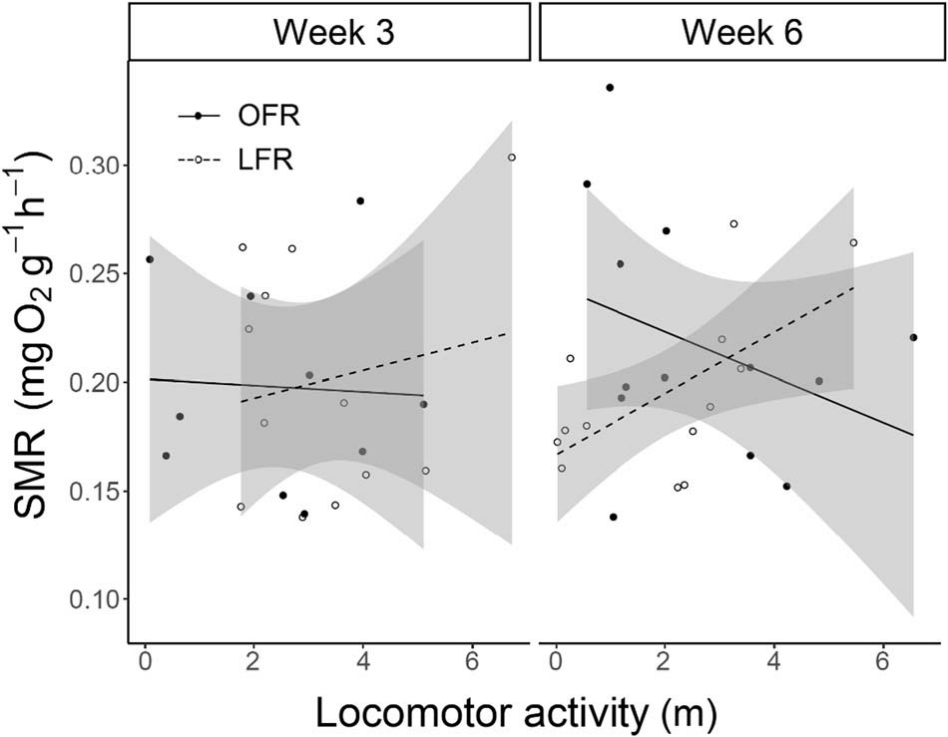
The relationship between SMR (mg O_2_ g^−1^ h^−1^) and locomotor activity of juvenile white sturgeon (*A. transmontanus*) reared at 15°C and fed OFR (closed circles and solid line) and LFR (50% of OFR, open circles and dashed line). The significant linear least squares regression for LFR fish at 6 weeks is represented by: locomotor activity = 0.167 + 0.014 (SMR), *P* = 0.028.

## Discussion

### Feed rate, SMR and locomotor activity

Only fish under the greatest nutritional stress (6 weeks at LFR) showed a significant relationship between SMR and locomotor activity (Fig. 4). These results suggest that while metabolic demand can play a role in determining behaviour, the extent of these effects may be context-dependent and vary, e.g. with factors such as the level of nutritional stress (i.e. 3 vs 6 weeks; see Killen *et al.,* 2011). LFR fish with relatively higher SMRs compared to other LFR fish may have engaged in increased activity in attempts to acquire food for maintaining their more metabolically demanding machinery. This supports findings by Killen *et al.* (2011) in which juvenile European sea bass (*D. labrax*) deprived of food for 7 days revealed a significant positive correlation between metabolic rate and activity (measured as the number of transitions between one covered and three open areas of a raceway) when it did not exist prior to food deprivation. Additionally, Killen *et al.* (2011) found a significant positive correlation between instantaneous mass loss and metabolic rate, further supporting that relatively higher SMR values require more energy to maintain. Furthermore, there were among-individual differences in behavioural changes pre- and post-food deprivation. Our study used different individuals between the 3- and 6-week measurements, revealing overall trends but not consistent individual differences in the effect of nutritional stress on the relationship between SMR and locomotor activity. This is a particularly interesting avenue for future study, as it is thought that consistent individual differences in physiological state are an important factor that promote the formation of individual differences in personality (Biro and Stamps, 2008; Houston, 2010; Mittelbach *et al.,* 2014).

While the effect of nutritional stress on SMR and locomotor activity is relatively subtle in this study, optimal feed rates are laboratory-based rations for maximum growth and not representative of all conditions experienced by fish in the wild. We knew our feed rates were sufficient to create stunted growth between the OFR and LFR fish (Poletto *et al.,* 2018), but the lack of wild juvenile white sturgeon monitoring or diet studies limits comparison to actual field feed rates. It is likely that wild white sturgeon experience fluctuations in food availability that have the capacity to influence behaviour on a more consistent and extreme basis than seen here, such that our LFR ration likely represents a greater food availability than typical wild diets. Additionally, body size may affect these behavioural responses, as smaller fish are at greater risk of predation and their higher mass-specific metabolic rates make them more prone to starvation (Post and Evans, 1989).

### SMR and feed rate

Contrary to our prediction, LFR fish did not exhibit a reduced SMR relative to OFR fish at either 3 or 6 weeks (Fig. 3a), while whole-body *Ṁ*O_2_ behaved as expected, with OFR fish growing larger and thus having a greater quantity of respiring tissue at both timepoints (Fig. 3b, Fig. S1). A significant relationship between SMR (accounting for differences in mass) and ration level has been shown in coho salmon (*Oncorhynchus kisutch*) after 6 weeks (Van Leeuwen *et al.,* 2012), Atlantic salmon (*Salmo salar*) after 4 weeks (O’Connor *et al.,* 2000) and juvenile brown trout (*Salmo trutta*) after 4 weeks (Auer *et al.,* 2015). In brown trout, these shifts were additionally linked with differences in somatic growth, with individuals who had greater increases in SMR exhibiting faster growth (Auer *et al.,* 2015). These individual differences in response to food deprivation or food increases support the idea that consistent individual differences in SMR can be correlated with other physiological changes. Van Leeuwen *et al.* (2012) suggest that the reduction in SMR under food deprivation is likely due to the involuntary reduction in anabolic and catabolic processes associated with reduced food consumption and growth, rather than a voluntary suppression of metabolism due to low food. Interestingly, starvation physiology of rainbow trout (*Oncorhynchus mykiss*) showed that digestive somatic index declined during starvation, indicating a quicker mobilization of energy reserves from the digestive tract with respect to the overall body mass, while Adriatic sturgeon (*Acipenser naccarii*) mobilized energy from muscle and liver tissues (Furne and Sanz, 2018). Different tissues and organs show considerable variation in mass-specific metabolic rates, so nutritional stress with regards to SMR may present differently in sturgeon than in teleosts (Metcalfe *et al.,* 2023). Flexibility in energy metabolism is thought to be important for maximizing growth rates under challenges imposed by variable food availability, which is a common occurrence in nature for most fishes.

The lack of a response in SMR to low feed in our sturgeon, despite large differences in body size, was unexpected. Extending the time at ration may have resulted in significant differences, but evidence also suggests that sturgeon do not display physiological responses to stress typical to teleost species. For example, juvenile pallid sturgeon (*Scaphirhynchus albus*) and hybrid pallid $\times$ shovelnose (*S. albus*$\times$  *platorynchus*) exposed to a 30-s handling exposure failed to evoke increases in plasma cortisol, lactate or glucose, though a significant increase in plasma cortisol was elicited after a 6-h severe confinement stressor with handling (Barton *et al.,* 2000). In nocturnally active green sturgeon (*Acipenser medoristris*), time of day affected plasma cortisol and lactate, but not glucose, with increased cortisol and lactate in fish stressed at night. For Atlantic sturgeon (*Acipenser oxyrhynchus*) and shortnose sturgeon (*Acipenser brevirostrum*), *Ṁ*O_2_ values were 10–20% lower than teleosts of comparable size and post-exercise *Ṁ*O_2_ only increased 2-fold. Additionally, the physiological response to exercise subsided relatively rapidly compared to teleosts (Kieffer *et al.,* 2001). Feed restriction in green sturgeon fingerlings, however, did perturb metabolites related to energy metabolism, osmolality regulation and antioxidation capacity within kidney, liver and muscle tissues (Lin *et al.,* 2019). Altogether, this suggests that physiological changes resulting from nutritional stress in sturgeon are better studied using molecular techniques that can individually assess organ function rather than whole-body metrics such as SMR, and that primitive fishes should not be assumed to behave physiologically as teleosts do.

### Growth

Feed rate significantly affected length and wet mass after both 3 and 6 weeks at ration, which was expected as juvenile sturgeon exhibit rapid rates of growth (Fig. 1a and b). Body size has important implications for fish performance and survival in nature. In sympatric green sturgeon, predation decreases with increases in size (Baird *et al.,* 2020), while critical swimming velocity (Verhille *et al.,* 2014) and salinity tolerance (Allen *et al.,* 2011) increase with increasing size. Nutritional levels can also alter heat shock protein levels, affecting critical thermal maximum (Verhille *et al.,* 2016). In contrast, log–log transformed length and wet mass exhibited no difference in the rate of growth under each treatment, indicated by the slope of the length–weight relationship (Fig. 2). This tendency for similar growth trajectories in sturgeon is not fully understood but was also observed in larval and early juvenile green sturgeon (Lo *et al.,* 2024). As all fish for this study were from the same cohort, it is possible that parentage exerted some uniform influence on early-life growth and is an avenue for further study.

### Conclusion

Changing food web dynamics in the SSJRB requires species-specific investigation, especially with regards to the invasion of the overbite clam (*Potamocorbula amurensis*), which has drastically altered the benthic prey community. White sturgeon have been able to exploit this new invasive prey source, but the caloric value is low and selenium bioaccumulation may result in reproductive toxicity (Zeug *et al.,* 2014). Decreased species richness is expected to lead to less stable food webs, and an increased likelihood of prey collapse for white sturgeon.

The relationship between physiological traits and behaviour is an important aspect of individual response, as behavioural shifts are often the first response to a perturbation that an organism can undertake. These shifts in behaviour may be adaptive responses to increase the probability of survival and could assist in species-specific conservation strategies. Nutritional stress is a ubiquitous environmental stressor, and white sturgeon are just one of several declining anadromous fish species in the SSJRB that are likely to be increasingly impacted by continued food web changes. This study provides evidence that SMR and locomotor activity in white sturgeon juveniles exhibits a context-dependent response to sufficient nutritional stress. Assessing how other types of stressors may affect this relationship is an avenue of future study.

## Supplementary Material

Web_Material_coaf039

## Data Availability

The data needed to reproduce the statistical analyses and figures in this study are publicly archived on Figshare at https://doi.org/10.6084/m9.figshare.26196431.v1.

## References

[ref1] Allen PJ, McEnroe M, Forostyan T, Cole S, Nicholl MM, Hodge B, Cech JJ (2011) Ontogeny of salinity tolerance and evidence for seawater-entry preparation in juvenile green sturgeon, *Acipenser medirostris*. J Comp Physiol B 181: 1045–1062. 10.1007/s00360-011-0592-0. 21630040

[ref2] Álvarez D, Nicieza AG (2005) Is metabolic rate a reliable predictor of growth and survival of brown trout (*Salmo trutta*) in the wild? Can J Fish Aquat Sci 62: 643–649. 10.1139/f04-223.

[ref52] Armstrong, JD, Millidine, KJ, Metcalfe, NB (2011) Ecological consequences of variation in standard metabolism and dominance among salmon parr. Ecol Freshw Fish 20: 371–376. Portico. 10.1111/j.1600-0633.2011.00486.x.

[ref3] Auer SK, Salin K, Anderson GJ, Metcalfe NB (2016a) Flexibility in metabolic rate and activity level determines individual variation in overwinter performance. Oecologia 182: 703–712. 10.1007/s00442-016-3697-z. 27461377 PMC5043002

[ref4] Auer SK, Salin K, Rudolf AM, Anderson GJ, Metcalfe NB (2015) Flexibility in metabolic rate confers a growth advantage under changing food availability. J Anim Ecol 84: 1405–1411. 10.1111/1365-2656.12384. 25939669 PMC4682473

[ref5] Auer SK, Salin K, Rudolf AM, Anderson GJ, Metcalfe NB (2016b) Differential effects of food availability on minimum and maximum rates of metabolism. Biol Lett 12: 20160586. 10.1098/rsbl.2016.0586. 28120798 PMC5095193

[ref6] Baird SE, Steel AE, Cocherell DE, Poletto JB, Follenfant R, Fangue NA (2020) Experimental assessment of predation risk for juvenile green sturgeon, *Acipenser medirostris*, by two predatory fishes. J Appl Ichthyol 36: 14–24. 10.1111/jai.13990.

[ref7] Barton BA, Bollig H, Hauskins BL, Jansen CR (2000) Juvenile pallid (*Scaphirhynchus albus*) and hybrid pallid×shovelnose (*S. albus×platorynchus*) sturgeons exhibit low physiological responses to acute handling and severe confinement. Comp Biochem Physiol A Mol Integr Physiol 126: 125–134. 10.1016/S1095-6433(00)00192-6. 10908860

[ref8] Biro PA, Stamps JA (2008) Are animal personality traits linked to life-history productivity? Trends Ecol Evol 23: 361–368. 10.1016/j.tree.2008.04.003. 18501468

[ref9] Blackburn SE, Gingras ML, DuBois J, Jackson ZJ, Quist MC (2019) Population dynamics and evaluation of management scenarios for white sturgeon in the Sacramento–San Joaquin River Basin. North Am J Fish Manag 39: 896–912. 10.1002/nafm.10316.

[ref10] Brandl SJ, Lefcheck JS, Bates AE, Rasher DB, Norin T (2023) Can metabolic traits explain animal community assembly and functioning? Biol Rev 98: 1–18. 10.1111/brv.12892. 36054431

[ref11] Brown LR, Moyle PB (2005) Native fishes of the Sacramento–San Joaquin drainage, California: a history of decline. Am Fish Soc Symp 75–98.

[ref12] Burton T, Killen SS, Armstrong JD, Metcalfe NB (2011) What causes intraspecific variation in resting metabolic rate and what are its ecological consequences? Proc R Soc B Biol Sci 278: 3465–3473. 10.1098/rspb.2011.1778. PMC3189380 21957133

[ref13] California Fish and Game Commission . 2023. Item No. 9 White Sturgeon Emergency Regulation. Staff Summary for October 11–12, 2023. Author: Jenn Bacon. Available at: https://nrm.dfg.ca.gov/FileHandler.ashx?DocumentID=216457&inline

[ref14] Chabot D, Steffensen JF, Farrell AP (2016) The determination of standard metabolic rate in fishes: measuring SMR in fishes. J Fish Biol 88: 81–121. 10.1111/jfb.12845. 26768973

[ref15] Cohen AN, Carlton JT (1998) Accelerating invasion rate in a highly invaded estuary. Science 279: 555–558. 10.1126/science.279.5350.555. 9438847

[ref16] Deng D-F, Koshio S, Yokoyama S, Bai SC, Shao Q, Cui Y, Hung SSO (2003) Effects of feeding rate on growth performance of white sturgeon (*Acipenser transmontanus*) larvae. Aquaculture 217: 589–598. 10.1016/S0044-8486(02)00461-1.

[ref17] Fish MA (2010) A white sturgeon year-class index for the San Francisco estuary and its relation to delta outflow. IEP Newsletter 23: 80–84.

[ref18] Furne M, Sanz A (2018) Starvation in fish – sturgeon and rainbow trout as examples. In V Preedy, VB Patel, eds, Handbook of Famine, Starvation, and Nutrient Deprivation: From Biology to Policy. Springer International Publishing, Cham, pp. 1–16

[ref19] Houston AI (2010) Evolutionary models of metabolism, behaviour and personality. Philos Trans R Soc B Biol Sci 365: 3969–3975. 10.1098/rstb.2010.0161. PMC2992740 21078649

[ref20] Kamler E (2008) Resource allocation in yolk-feeding fish. Rev Fish Biol Fish 18: 143–200. 10.1007/s11160-007-9070-x.

[ref21] Kieffer J, Wakefield A, Litvak M (2001) Juvenile sturgeons exhibit low physiological responses to exercise. J Exp Biol 204: 4281–4289. 10.1242/jeb.204.24.4281. 11815652

[ref22] Killen SS, Glazier DS, Rezende EL, Clark TD, Atkinson D, Willener AST, Halsey LG (2016) Ecological influences and morphological correlates of resting and maximal metabolic rates across teleost fish species. Am Nat 187: 592–606. 10.1086/685893. 27104992

[ref23] Killen SS, Marras S, McKenzie DJ (2011) Fuel, fasting, fear: routine metabolic rate and food deprivation exert synergistic effects on risk-taking in individual juvenile European sea bass. J Anim Ecol 80: 1024–1033. 10.1111/j.1365-2656.2011.01844.x. 21790592

[ref24] Killen SS, Marras S, Metcalfe NB, McKenzie DJ, Domenici P (2013) Environmental stressors alter relationships between physiology and behaviour. Trends Ecol Evol 28: 651–658. 10.1016/j.tree.2013.05.005. 23756106

[ref25] Killen SS, Marras S, Ryan MR, Domenici P, McKenzie DJ (2012) A relationship between metabolic rate and risk-taking behaviour is revealed during hypoxia in juvenile European sea bass. Funct Ecol 26: 134–143. 10.1111/j.1365-2435.2011.01920.x.

[ref26] Lee S, Wang Y, Hung SSO, Strathe AB, Fangue NA, Fadel JG (2014) Development of optimum feeding rate model for white sturgeon (*Acipenser transmontanus*). Aquaculture 433: 411–420. 10.1016/j.aquaculture.2014.06.007.

[ref27] Lin C-Y, Huang L-H, Deng D-F, Lee S-H, Liang H-J, Hung SSO (2019) Metabolic adaptation to feed restriction on the green sturgeon (*Acipenser medirostris*) fingerlings. Sci Total Environ 684: 78–88. 10.1016/j.scitotenv.2019.05.044. 31150878

[ref28] Lo VK, Martin BT, Danner EM, Cocherell DE, Cech JJ Jr, Fangue NA (2022) The effect of temperature on specific dynamic action of juvenile fall-run Chinook salmon, *Oncorhynchus tshawytscha*. Conserv Physiol 10: coac067. 10.1093/conphys/coac067. 36325131 PMC9616469

[ref29] Lo VK, Zillig KW, Cocherell DE, Todgham AE, Fangue NA (2024) Effects of low temperature on growth and metabolism of larval green sturgeon (*Acipenser medirostris*) across early ontogeny. J Comp Physiol B 194: 427–442. 10.1007/s00360-024-01568-y. 38955877 PMC11316702

[ref30] Metcalfe NB, Bellman J, Bize P, Blier PU, Crespel A, Dawson NJ, Dunn RE, Halsey LG, Hood WR, Hopkins M et al. (2023) Solving the conundrum of intra-specific variation in metabolic rate: a multidisciplinary conceptual and methodological toolkit: new technical developments are opening the door to an understanding of why metabolic rate varies among individual animals of a species. Bioessays 45: 2300026. 10.1002/bies.202300026. 37042115

[ref31] Metcalfe NB, Taylor AC, Thorpe JE (1995) Metabolic rate, social status and life-history strategies in Atlantic salmon. Anim Behav 49: 431–436. 10.1006/anbe.1995.0056.

[ref32] Metcalfe NB, Van Leeuwen TE, Killen SS (2016) Does individual variation in metabolic phenotype predict fish behaviour and performance? Effects of variation in metabolic rate. J Fish Biol 88: 298–321. 10.1111/jfb.12699. 26577442 PMC4991269

[ref33] Mittelbach GG, Ballew NG, Kjelvik MK (2014) Fish behavioral types and their ecological consequences. Can J Fish Aquat Sci 71: 927–944. 10.1139/cjfas-2013-0558.

[ref34] Moyle PB, Kiernan JD, Crain PK, Quiñones RM (2013) Climate change vulnerability of native and alien freshwater fishes of California: a systematic assessment approach. PloS One 8: e63883. 10.1371/journal.pone.0063883. 23717503 PMC3661749

[ref35] Mueller P, Diamond J (2001) Metabolic rate and environmental productivity: well-provisioned animals evolved to run and idle fast. Proc Natl Acad Sci 98: 12550–12554. 10.1073/pnas.221456698. 11606744 PMC60091

[ref36] Nespolo RF, Franco M (2007) Whole-animal metabolic rate is a repeatable trait: a meta-analysis. J Exp Biol 210: 2000–2005. 10.1242/jeb.02780. 17515425

[ref37] O’Connor KI, Taylor AC, Metcalfe NB (2000) The stability of standard metabolic rate during a period of food deprivation in juvenile Atlantic salmon. J Fish Biol 57: 41–51. 10.1111/j.1095-8649.2000.tb00774.x.

[ref38] Poletto JB, Martin B, Danner E, Baird SE, Cocherell DE, Hamda N, Cech JJ Jr, Fangue NA (2018) Assessment of multiple stressors on the growth of larval green sturgeon *Acipenser medirostris*: implications for recruitment of early life-history stages. J Fish Biol 93: 952–960. 10.1111/jfb.13805. 30246375

[ref39] Post JR, Evans DO (1989) Size-dependent overwinter mortality of young-of-the-year yellow perch (*Perca flavescens*): laboratory, in situ enclosure, and field experiments. Can J Fish Aquat Sci 46: 1958–1968. 10.1139/f89-246.

[ref40] Sogard SM (1997) Size-selective mortality in the juvenile stage of teleost fishes: a review. Bull Mar Sci 60: 1129–1157.

[ref41] Sommer T, Armor C, Baxter R, Breuer R, Brown L, Chotkowski M, Culberson S, Feyrer F, Gingras M, Herbold B et al. (2007) The collapse of pelagic fishes in the Upper San Francisco Estuary. Fisheries 32: 270–277. 10.1577/1548-8446(2007)32[270:TCOPFI]2.0.CO;2.

[ref42] Speakman JR, Król E, Johnson MS (2004) The functional significance of individual variation in basal metabolic rate. Physiol Biochem Zool 77: 900–915. 10.1086/427059. 15674765

[ref43] Stompe DK, Moyle PB, Kruger A, Durand JR (2020) Comparing and integrating fish surveys in the San Francisco Estuary: why diverse long-term monitoring programs are important. San Franc Estuary Watershed Sci 18. 10.15447/sfews.2020v18iss2art4.

[ref44] Svendsen MBS, Bushnell PG, Steffensen JF (2016) Design and setup of intermittent-flow respirometry system for aquatic organisms. J Fish Biol 88: 26–50. 10.1111/jfb.12797. 26603018

[ref45] Van Eenennaam JP, Linares-Casenave J, Doroshov SI (2012) Tank spawning of first generation domestic green sturgeon. J Appl Ichthyol 28: 505–511. 10.1111/j.1439-0426.2012.02012.x.

[ref46] Van Leeuwen TE, Rosenfeld JS, Richards JG (2012) Effects of food ration on SMR: influence of food consumption on individual variation in metabolic rate in juvenile coho salmon (*Onchorhynchus kisutch*). J Anim Ecol 81: 395–402. 10.1111/j.1365-2656.2011.01924.x. 22066987

[ref47] Verhille CE, Lee S, Todgham AE, Cocherell DE, Hung SSO, Fangue NA (2016) Effects of nutritional deprivation on juvenile green sturgeon growth and thermal tolerance. Environ Biol Fishes 99: 145–159. 10.1007/s10641-015-0463-8.

[ref48] Verhille CE, Poletto JB, Cocherell DE, DeCourten B, Baird S, Cech JJ, Fangue NA (2014) Larval green and white sturgeon swimming performance in relation to water-diversion flows. Conserv Physiol 2. 10.1093/conphys/cou031. PMC4806727 27293652

[ref49] Yoshiyama RM, Fisher FW, Moyle PB (1998) Historical abundance and decline of Chinook salmon in the Central Valley region of California. North Am J Fish Manag 18: 487–521. 10.1577/1548-8675(1998)018<0487:HAADOC>2.0.CO;2.

[ref50] Zarri LJ, Palkovacs EP (2019) Temperature, discharge and development shape the larval diets of threatened green sturgeon in a highly managed section of the Sacramento River. Ecol Freshw Fish 28: 257–265. 10.1111/eff.12450.

[ref51] Zeug S, Brodsky A, Kogut N, Stewart A, Merz J (2014) Ancient fish and recent invaders: white sturgeon Acipenser transmontanus diet response to invasive-species-mediated changes in a benthic prey assemblage. Mar Ecol Prog Ser 514: 163–174. 10.3354/meps11002.

