# Supplementary material for: The role of nutritional state in the relationship between standard metabolic rate and locomotor activity in juvenile white sturgeon (Acipenser transmontanus), with implications for anthropogenically altered food webs
Source: Conserv Physiol. 2025 Jun 12;13(1):coaf039. doi: 10.1093/conphys/coaf039 (PMC12162131; doi:10.1093/conphys/coaf039)

The role of nutritional state in the relationship between standard metabolic rate and locomotor activity in juvenile white sturgeon (*Acipenser transmontanus*), with implications for anthropogenically-altered food webs

Figure S1: Whole-body metabolic rate (mg O_2 h^{-1}) regressed against wet mass for juvenile White Sturgeon (*Acipenser transmontanus*) reared at 15°C fed optimal feed ration (OFR, closed circles and solid line) and low feed ration (LFR, 50% of OFR, open circles and dashed line), plotted on log-log transformed axes. Lines represent least squares regressions and gray ribbons represent standard error of the fit.

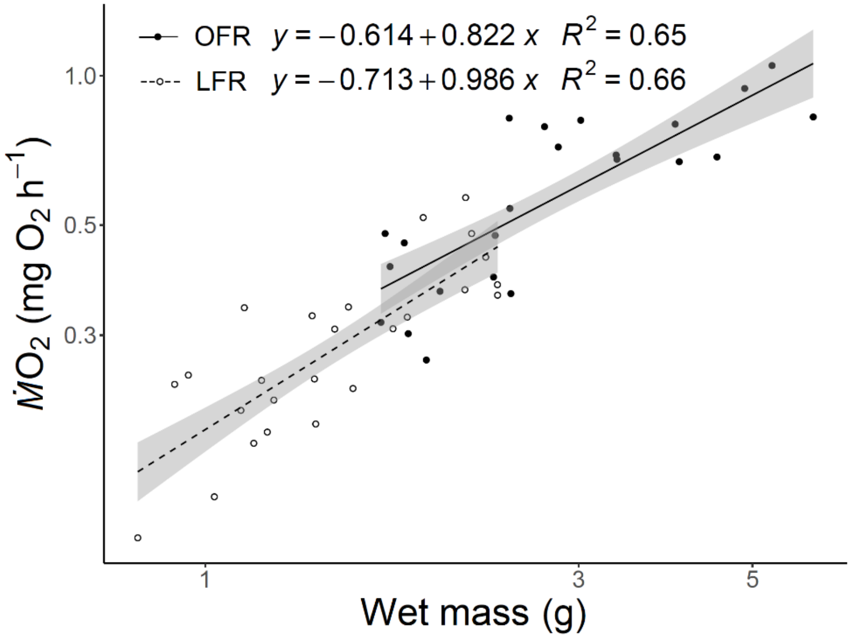

Supplement: Web_Material_coaf039 [file web_material_coaf039.zip › Lo_Supplementary_Figure.pdf]
